# A Rare Case of Necrotizing Fasciitis Presenting As Tarsal Tunnel Syndrome in a Patient With Uncontrolled Diabetes

**DOI:** 10.7759/cureus.75339

**Published:** 2024-12-08

**Authors:** Janak Parmar

**Affiliations:** 1 Trauma and Orthopedics, Lister Hospital, Stevenage, United Kingdom

**Keywords:** diabetic, fasciectomy, flexor retinaculum, necrotizing fasciitis, tarsal tunnel syndrome

## Abstract

Necrotizing fasciitis is a severe and rapidly progressing soft tissue infection that requires immediate intervention. However, its manifestation as tarsal tunnel syndrome in a diabetic patient is an extremely rare occurrence, with no previous reports found in the existing literature. We present a case report of a patient in their late 50s with uncontrolled diabetes who had necrotizing fasciitis and presented initially to the emergency department with hypotension. The patient complained of symptoms consistent with acute tarsal tunnel syndrome and displayed systemic signs of infection, necessitating urgent surgical intervention. Intraoperatively, the necrotic fascia involved the flexor retinaculum and its septa. A complete release of the tarsal tunnel and subsequent fasciectomy were performed. The patient experienced a successful postoperative recovery. This case emphasizes the importance of early recognition and intervention due to the aggressive nature of necrotizing fasciitis. Moreover, it highlights a unique presentation not previously reported in the literature, thereby contributing to increased awareness of this rare phenomenon in diabetic patients.

## Introduction

Necrotizing fasciitis is a life-threatening soft tissue infection characterized by rapid progression, high morbidity, and mortality rates. It involves necrosis of fascia by fulminating polymicrobial infection, leading to tissue necrosis and systemic toxicity. Delay in intervention is associated with poor outcomes. Diabetes mellitus, malignancy, and other immunosuppressive conditions are some of the risk factors [1]. Diagnosing necrotizing fasciitis is challenging due to the lack of specific signs, which can delay treatment [2]. Shock, advanced age, and diabetes are associated with a poor prognosis [3].

Although the lower extremities are commonly affected, necrotizing fasciitis presenting as tarsal tunnel syndrome in a diabetic patient is exceptionally rare. We present a unique case of necrotizing fasciitis initially masquerading as tarsal tunnel syndrome in an uncontrolled diabetic patient [4]. 

## Case presentation

**Figure 1:**
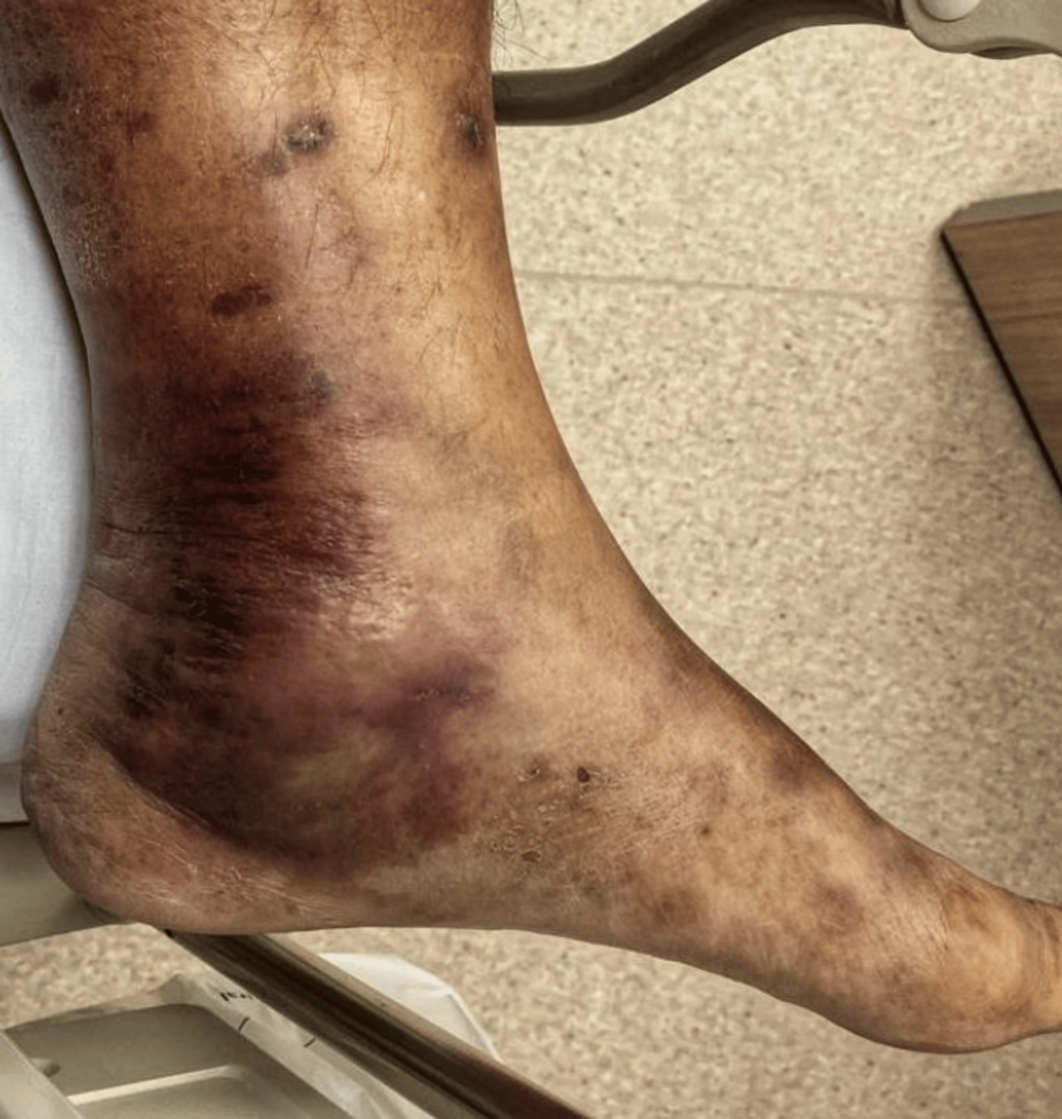
Clinical presentation of necrotizing fasciitis at the tarsal tunnel

A 57-year-old patient with a known history of uncontrolled diabetes mellitus presented to the emergency department with hypotension and reported symptoms consistent with necrotizing fasciitis. The patient complained of sudden onset persistent burning pain and paresthesia in the sole along the tibial nerve and both plantar cutaneous nerves, suggestive of acute tarsal tunnel syndrome [5]. Physical examination revealed erythema, warmth, swelling along the tarsal tunnel, and systemic signs of infection (Figure 1) [6]. The patient had high C-reactive protein (253 mg/dL), white cell count (18 thousand/uL), raised urea (16 mmol/L), and creatinine (180 umol/L).

**Figure 2:**
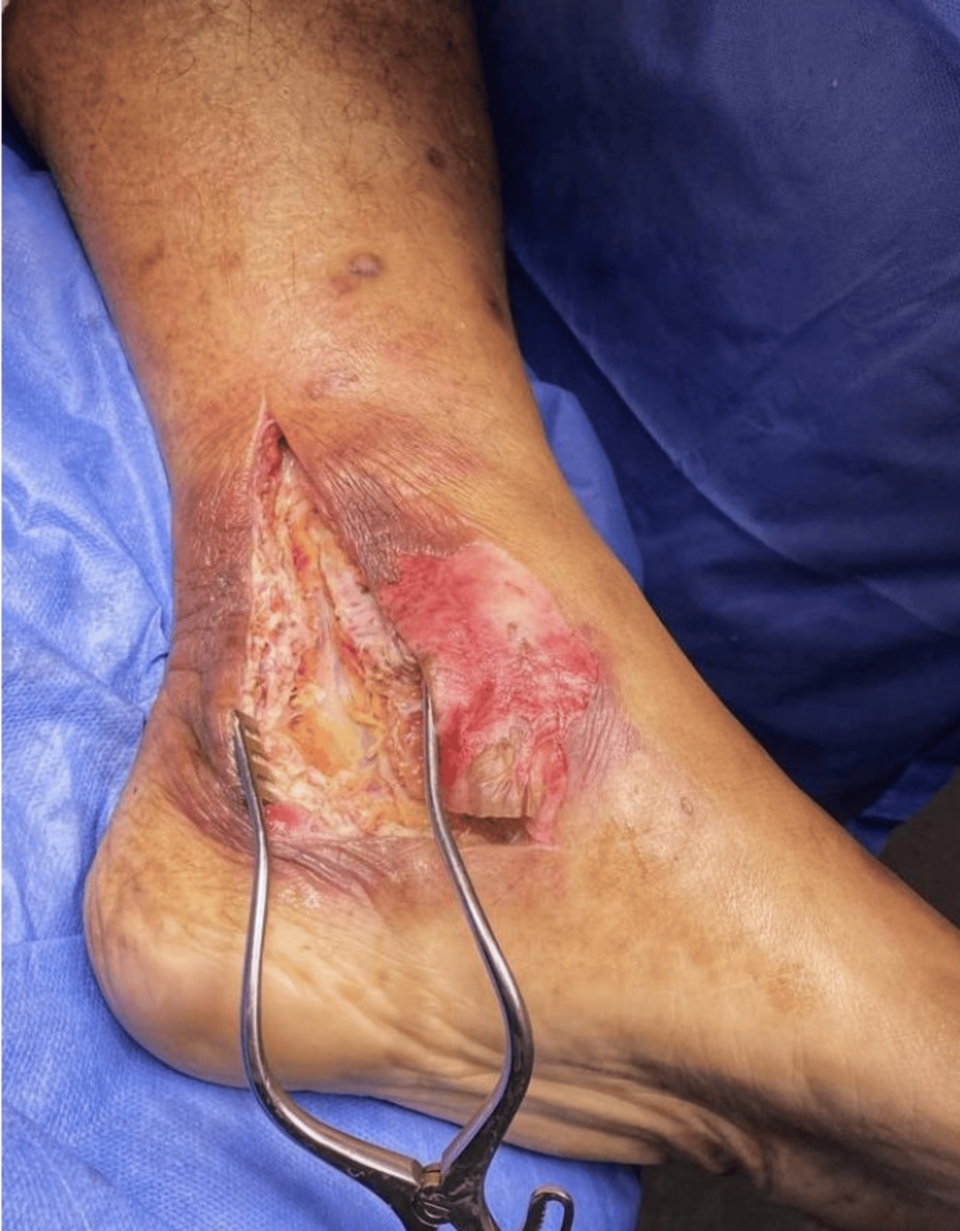
After surgical debridement of the tarsal tunnel

After the initial resuscitation, due to the morbidity and mortality associated with necrotizing fasciitis, the patient was promptly taken to the operating room. The patient was started on broad-spectrum antibiotics, which is a combination of Meropenem 1 g IV every eight hours, vancomycin 20 mg/kg/dose every eight hours, and clindamycin 600 mg four times daily. Intraoperatively, the necrotic fascia involved the flexor retinaculum and its septa. There was the presence of dishwater-like fluid at the opening of the retinaculum. A thorough release of the entire tarsal tunnel was performed to relieve compression of the posterior tibial nerve. Subsequently, a fasciectomy was carried out to remove all necrotic tissue (Figure 2). The surgical procedure was successful, and the patient was transferred to the high-dependency unit for further management.

During the postoperative period, the patient received appropriate wound care, intravenous antibiotics in consultation with an infectious disease specialist and microbiologist, and supportive therapy for glycemic control. The intraoperative culture grew staphylococcus aureus, and antibiotics were started as per the sensitivities [7]. The patient underwent two further debridements. Plastic surgeons did wound coverage with vacuum-assisted closure and skin grafting [8]. The patient improved significantly postoperatively in two days, resolving pain, erythema, swelling, and paresthesia. Follow-up examinations showed no signs of infection, and the patient's foot function gradually improved over time.

## Discussion

Necrotizing fasciitis is a largely aggressive infection with a high mortality rate if not diagnosed in time. Necrotizing Fascitis manifests as soft-tissue edema, erythema, severe pain out of proportion to signs, tenderness, and skin bullae or necrosis, most of which were present in our patient [9]. This can easily be confused with soft-tissue infections such as cellulitis, impetigo, erysipelas, and early abscesses. Our case manifested features of acute tarsal tunnel syndrome along with infection [10].

Tarsal tunnel syndrome is usually associated with nerve entrapment rather than infection. Patients with tarsal tunnel syndrome present with gradual onset paresthesia, numbness, and burning pain in the sole, which worsens with weight-bearing and is relieved when the foot is placed in an elevated position [11,12]. Our patient had these features, but they were of sudden onset, and the pain was continuous without any relief. The pain was along the tibial nerve and both plantar nerves.

Immediate surgical intervention allowed for the immediate removal of necrotic tissue, preventing further tissue destruction. The high degree of suspicion and timely diagnosis is essential. Considering the urgency of the situation, the diagnosis was mainly clinical, and the patient was operated on within four hours of the visit to Accident and Emergency. Given the urgency of the situation, we did not do other investigations like MRI. Our patient had a culture that grew Staphylococcus aureus, one of the organisms known to cause necrotizing fasciitis. Blood parameters were consistent with a high LRINEC score of nine [13]. However, the diagnosis of necrotizing fasciitis is mainly clinical.

Necrotizing fasciitis is classically associated with high morbidity and mortality. Our patient survived due to prompt diagnosis and emergency surgery with proper antibiotics and wound coverage. 

## Conclusions

Awareness of this rare phenomenon can aid healthcare providers in timely diagnosis and management, ultimately leading to better patient care and outcomes. This case report highlights the importance of maintaining a high degree of suspicion, early recognition, and timely intervention in cases of necrotizing fasciitis, particularly in diabetic patients. A multidisciplinary approach to managing such patients is crucial in reducing mortality and morbidity.
